# Self-collection of capillary blood and saliva to determine COVID-19 vaccine immunogenicity in patients with immune-mediated inflammatory diseases and health professionals

**DOI:** 10.3389/fpubh.2022.994770

**Published:** 2022-10-14

**Authors:** Caroline Schmetzer, Ekaterina Vogt, Laura Stellar, Elie-Tino Godonou, Anna-Maria Liphardt, Felix Muehlensiepen, Nicolas Vuillerme, Axel J. Hueber, Arnd Kleyer, Gerhard Krönke, Georg Schett, David Simon, Johannes Knitza

**Affiliations:** ^1^Department of Internal Medicine 3 – Rheumatology and Immunology Friedrich-Alexander University Erlangen-Nürnberg and Universitätsklinikum Erlangen, Erlangen, Germany; ^2^Deutsches Zentrum für Immuntherapie, Friedrich-Alexander University Erlangen-Nürnberg and Universitätsklinikum Erlangen, Erlangen, Germany; ^3^Thermo Fisher Scientific, Freiburg, Germany; ^4^Centre for Health Services Research Brandenburg, Brandenburg Medical School, Neuruppin, Germany; ^5^Faculty of Health Sciences Brandenburg, Brandenburg Medical School, Neuruppin, Germany; ^6^Université Grenoble Alpes, AGEIS, Grenoble, France; ^7^Institut Universitaire de France, Paris, France; ^8^LabCom Telecom4Health, Orange Labs and Univ. Grenoble Alpes, CNRS, Inria, Grenoble INP-UGA, Grenoble, France; ^9^Division of Rheumatology, Klinikum Nürnberg, Paracelsus Medical University, Nürnberg, Germany

**Keywords:** self-collection, capillary blood, remote care, telehealth, self-sampling, COVID-19

## Abstract

**Introduction:**

Being able to independently determine vaccine induced antibody responses by minimal-invasive methods is of great interest to enable a flexible and effective vaccination strategy. This study aimed to evaluate (1) the accuracy, feasibility, usability and acceptability of capillary blood and saliva self-sampling to determine SARS-CoV-2 antibody responses in patients with immune-mediated inflammatory diseases (IMIDs) and health professionals (HP).

**Methods:**

IMID patients and HP having received two doses of SARS-CoV-2 vaccines, self-collected capillary blood (Tasso+) and saliva samples. Capillary samples were considered interchangeable with venous blood if three criteria were met: Spearman's correlation coefficient (r) > 0.8, non-significant Wilcoxon signed-rank test (i.e., *p* > 0.05), and a small bias or 95% of tests within 10% difference through Bland-Altman. Participants completed a survey to investigate self-sampling usability (system usability scale; SUS) and acceptability (net promoter score; NPS). Study personnel monitored correct self-sampling completion and recorded protocol deviations.

**Results:**

60 participants (30 IMID patients and 30 HP) were analyzed. We observed interchangeability for capillary samples with an accuracy of 98.3/100% for Anti-SARS-CoV-2 IgG/IgA antibodies, respectively. Fifty-eight capillary blood samples and all 60 saliva samples were successfully collected within the first attempt. Usability of both self-sampling procedures was rated as excellent, with significantly higher saliva ratings (*p* < 0.001). Capillary self-sampling was perceived as significantly (*p* < 0.001) less painful compared to traditional venous blood collection. Participants reported a NPS for capillary and saliva self-sampling of +68% and +63%, respectively. The majority of both groups (73%) preferred capillary self-sampling over professional venous blood collection.

**Conclusion:**

Our results indicate that capillary self-sampling is accurate, feasible and preferred over conventional venous blood collection. Implementation could enable easy access, flexible vaccination monitoring, potentially leading to a better protection of vulnerable patient groups. Self-collection of saliva is feasible and safe however more work is needed to determine its application in clinical practice.

## Introduction

Evaluation of an adequate vaccination response and appropriate revaccinations are essential to counteract waning of humoral immune response (1) and to ensure a sustained and adequate level of protection (2, 3). Repeated measurement of anti-SARS-CoV-2 antibody levels is recommended especially for vulnerable patient groups, such as patients with immune-mediated inflammatory diseases (IMIDs) receiving immunsuppressive treatments, likely to have a poor vaccination response and to suffer from a severe COVID-19 infection (4). Due to the already limited number of available health professionals (HP) treating IMID patients (5), HP should try to prevent COVID-related absences, that can be avoided or shortened by maintaining an adequate vaccine immunogenicity.

Ideally, samples to investigate vaccine immunogenicity could be self-collected at home, and having to travel to healthcare facilities including the burden and infection risk, would be obsolete. Self-sampling enables independent, flexible collection of specimen, such as capillary blood (6) and saliva at home. Nwankwo et al. recently demonstrated how remote capillary blood self-sampling provides accurate results for several biomarkers, can improve shared decision making and overall patient experience (7). In a previous randomized controlled trial we showed that patients suffering from rheumatoid arthritis clearly preferred upper arm-based self-sampling with a self-adhesive lancet-based device (Tasso) to traditional finger pricking (8). Furthermore, a recent pilot study demonstrated that this upper-arm device (Tasso+) can be used by healthy and previously infected individuals to reliably collect blood for COVID-19 humoral response evaluation (9). Saliva represents a non-invasive and painless alternative to blood. Recent publications support the accuracy of saliva-based humoral response analysis (10–12). This saliva-based approach enabled a population-based Anti-SARS-CoV-2 antibody study in children, that might otherwise have been reluctant to conventional venous blood collection (11).

To the best of our knowledge, no study has yet directly compared capillary and saliva self-sampling in IMID patients and HP. Therefore, this study aimed to evaluate the accuracy, feasibility, usability and acceptability of capillary blood and saliva self-sampling to determine Anti-SARS-CoV-2 antibody responses in IMID patients and HP.

## Materials and methods

### Study design

**Figure 1:**
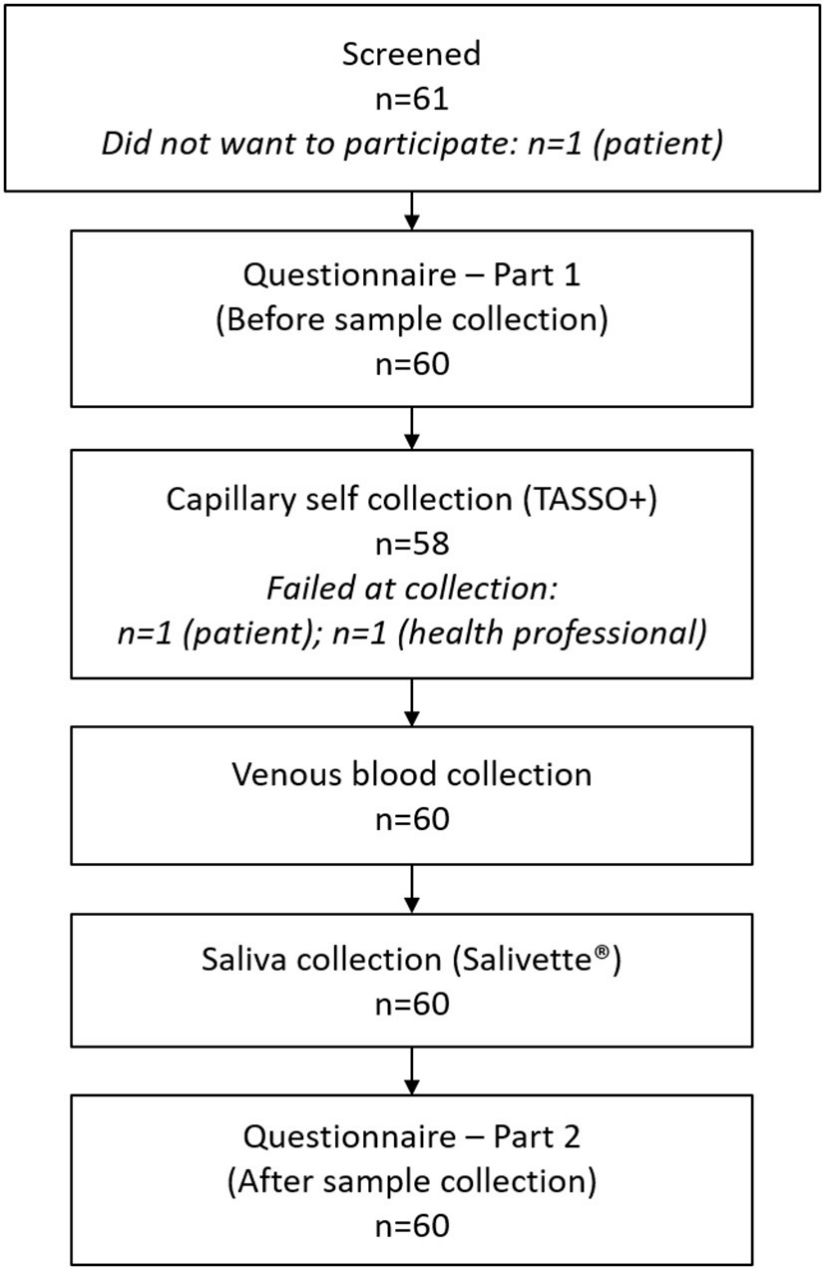
Participant flowchart.

This study was a prospective, single-center, cross-sectional, matched case-control study (WHO International Clinical Trials Registry: DRKS00024787), see Figure 1. Adult IMID patients were consecutively recruited at the outpatient clinic of the Department of Internal Medicine 3 (FAU Erlangen-Nurnberg) between May 2021 and August 2021. Patients were matched with local health professional controls (physicians and nurses), individually matched by same age and sex. The trial was approved by the local ethics authorities (Reg no. 25_21B) and written informed consent was obtained from all study participants. To be included, participants had to have received two doses of SARS-CoV-2 vaccine.

Participants first completed a questionnaire querying previous self-sampling experience and current attitude. After receiving written instructions, participants independently completed an upper-arm-based capillary and saliva specimen collection under the supervision of local study personnel. Additionally patients were presented a video instruction for the capillary self-sampling device. Deviations from the respective self-sampling protocol were recorded. After a traditional venous blood collection, representing the gold-standard, participants completed a final questionnaire to investigate perceived pain during blood collection and a potentially changed attitude toward self-sampling.

The agreement of anti-SARS-CoV-2 IgG and IgA antibody levels between matched capillary, saliva and venous samples was the primary outcome. Feasibility was assessed by the number of successfully collected samples within the first attempt. Usability of sampling devices was assessed via the ten-item System Usability Scale (SUS) (13). SUS scores range between zero (worst) and 100 (best). A score >68 is considered above average and a score >80 as high (13). Additionally, SUS scores were translated to categories such as “excellent” as previously described by Bangor et al. (14). The Net Promoter Score (NPS) (15, 16) was used to investigate acceptability after sample collection. Participants were queried how likely they are to recommend the self-sampling device to a friend or patient on a 11-point numeric rating scale (zero-not at all likely to 10-extremely likely). Answers between 0– and 6 are categorized as detractors, 7–8 as passives and 9–10 as promoters. The NPS is calculated by subtracting the percentage of detractors from the percentage of promoters. Participants were asked before and after sample collection “I would prefer capillary self-sampling instead of having to see a professional for a traditional venous blood collection” and report their level of agreement (strongly disagree to strongly agree). Pain perception of capillary self-sampling and venipuncture was measured using a 11-point numeric rating-scale (NRS; zero no pain at all, 10 worst imaginable pain) (17) directly after blood collection.

### Sample collection and processing

**Figure 2:**
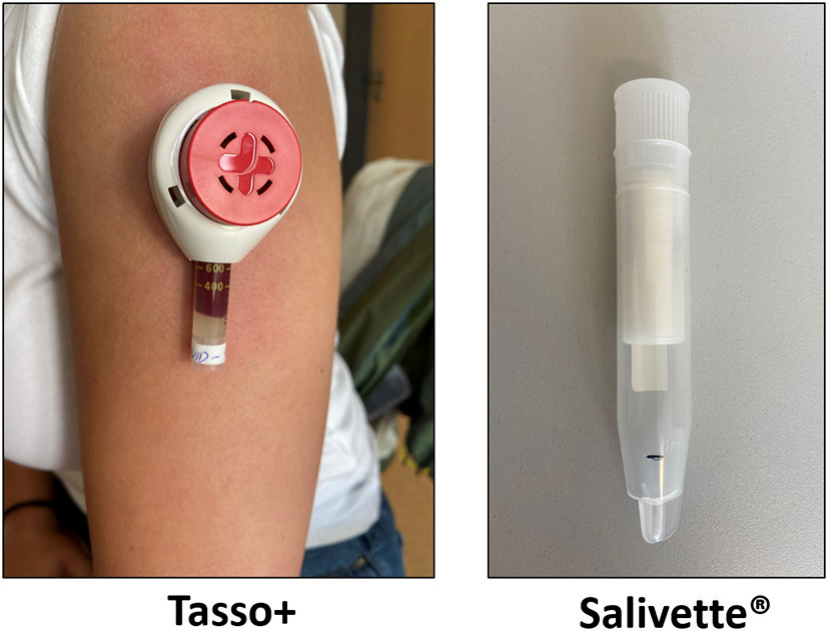
Capillary blood upper-arm self-collection device (Tasso+) and saliva collection device (Salivette^®^).

Capillary samples were collected using the upper-arm based Tasso+ device (Tasso Inc., Seattle, WA, USA) and saliva samples were collected using Salivetten Cotton Swab (Sarstedt AG & Co. KG, Nürmbrecht, Germany) by spitting directly into the tube without utilizing the cotton swab (Figure 2).

The Tasso+ device is attached to the upper arm by an adhesive and the lancet is activated by pressing a button. Prior to capillary blood collection, patients were instructed to warm the chosen collection site for 1 min by applying a heat-pad (L x W x H) 135 x 95 x 25 mm, max. heat 55°C, (Conrad Electronics SE, Germany) to increase local blood flow. Blood is then automatically collected using a vacuum. Participants were instructed to remove the device after a maximum collection time of 5 min or as soon as the collection tube was entirely filled with blood. Participants were instructed to collect a target volume of saliva up to a line mark. Participants should not drink or eat 30 min prior to saliva collection. Matched venous blood samples were collected by trained phlebotomists from all participants within 30 min of capillary blood and saliva collection.

Uncentrifuged capillary samples and centrifuged venous blood reference samples were sent by regular mail using standard postage and UN3373 compliant packaging to Thermo Fisher Scientific research laboratory in Freiburg, Germany. Samples were inspected independently by two lab technicians for quality. Upon arrival in the laboratory the samples were processed, resulting serum was transferred into Sarstedt™ 2 ml Polypropylene Micro Tubes (Sarstedt AG & Co., Nümbrecht, Germany) and stored at −20°C until analysis. Saliva samples were stored at the hospital at −20°C and then sent to Thermo Fisher Scientific research laboratory in Freiburg, Germany on dry ice and stored at −20°C until analysis. Prior to testing saliva samples were transferred to a new salivette tube so that all liquid was absorbed by the cotton pouch, followed by a 5 min, 4°C, 3,000 g centrifugation step. The eluate was collected and stored at −20°C. Saliva samples with ≥100 μl eluate volume were suitable for measurement on a Phadia 250 System.

Serum and saliva samples were tested on the Phadia 250 instrument platform (ThermoFisher Scientific, Phadia AB, Uppsala, Sweden). SARS-CoV-2 Spike 1 (S1) antigen (amino acid 14-681, expressed in mammalian cells) was adsorbed onto irradiated polystyrene EliA^TM^ wells and processed (18, 19). An additional test was developed to detect the IgA isotypes of anti-SARS-CoV-2 Spike 1 antibodies on the EliA™ instrument platform. For both, the EliA^TM^ SARS-CoV-2-Sp1 IgG and the test for IgA isotypes, values above 10 U/ml were considered to be reactive. No measurable correlation of results in the respective immunoglobulin subclass between saliva and corresponding serum samples were observed. Further measurements in saliva were discontinued.

### Statistical analysis

We adopted the sample size of previous self-sampling studies (9, 12) and did not perform a power calculation. These studies followed the FDA/EUA recommendation of 30 participants per group (12) and Green's rule of thumb calculation (20) for a linear regression for medium effect size and a minimum of 58 subjects (9).

Study group characteristics were summarized using appropriate descriptive statistics. Agreement between the two blood collection methods was assessed using a combination of three tests: Paired Wilcoxon signed rank test, correlation analysis, and Bland-Altman analysis. Clinical interchangeability between the two methods was a priori defined following the methodology by Nwankwo et al. (7): Non-significant paired Wilcoxon signed rank test, Spearman correlation coefficient >0.8, and small bias or max 10% difference between capillary and venous test results on Bland-Altman analysis. Bland-Altman limits of agreement were plotted and estimated. “Bias” is the average of the differences between the two methods of blood sampling, expressed as a percentage %. Spearman's correlation coefficient was calculated and plotted. Significance level was set as *p* < 0.05 for all statistical tests. The distribution of the pairs of variables, and of the difference between two pairs of variables, was assessed with normality tests (Shapiro-Wilk-Test, quantile-quantile plot). When the distribution of the pairs of variables did not follow a Gaussian distribution, non-parametric statistical tests were applied (Paired Wilcoxon signed rank test, Spearman's correlation). The Wilcoxon signed-rank test was used to compare the System Usability total Score (SUS) between capillary and saliva self-sampling and within the groups (patients and health professionals), when the assumptions for a paired *t*-test were not met. All analyses were completed using the R software environment (R version 4.1.1).

## Results

### Participants

A total of 61 participants (31 IMID patients, 30 HP) were screened for eligibility (Figure 1). One patient declined to participate, so that a total of 30 sex- and age-matched IMID and HP participants were included, Table 1. About 24/30 (80.0%) of IMID patients were receiving immunosuppressive treatment, most frequently biologic disease-modifying antirheumatic drugs (bDMARDs), 15 (50%), conventional synthetic disease-modifying antirheumatic drugs (csDMARDs), six (10.0%), and targeted synthetic DMARDs (tsDMARDs), three (10.0%). The most common IMIDs investigated were rheumatoid arthritis and psoriatic arthritis. The majoritiy of participants had received mRNA-based vaccines.

**Table 1:** Participant demographics.

**Parameter**	**Total** **(*n =* 60)**	**Patients** **(*n =* 30)**	**Health professionals** **(*n =* 30)**
Age, years, mean ± SD	49.4 ± 12.4	49.7 ± 12.2	49.0 ± 12.7
Female, *n* (%)	46 (76.7)	23 (76.7)	23 (76.7)
BMI, kg/m^2^, mean ± SD	25.7 ± 5.1	26.2 ± 5.4	25.3 ± 4.8
Previous self-sampling experience, *n* (%)	18 (30.0)	8 (26.7)	10 (33.3)
Previous saliva-sampling experience, *n* (%)	21 (35.0)	10 (33.3)	11 (36.7)
Actively smoking	14 (23.3)	7 (23.3)	7 (23.3)
**Diagnosis**, ***n*** **(%)**			
Rheumatoid arthritis	9 (15.0)	9 (30.0)	–
Psoriatic arthritis	9 (15.0)	9 (30.0)	–
Polymyalgia rheumatica	1 (1.7)	1 (3.3)	–
Systemic lupus erythematosus	1 (1.7)	1 (3.3)	–
Axial spondyloarthritis	3 (5.0)	3 (10.0)	–
Microscopic polyangiitis	1 (1.7)	1 (3.3)	–
Psoriasis	1 (1.7)	1 (3.3)	–
Crohn's disease	2 (3.3)	2 (6.7)	–
Anti-synthetase syndrome	1 (1.7)	1 (3.3)	–
Ulcerative colitis	2 (3.3)	2 (6.7)	–
**Education status**, ***n*** **(%)**			
High School graduate	35 (58.3)	18 (60.0)	17 (56.7)
College graduate	14 (23.3)	7 (23.3)	7 (23.3)
University graduate	11 (18.3)	5 (16.7)	6 (20.0)
**Treatment**			
No treatment	36 (60.0)	6 (20.0)	30 (100.0)
bDMARDs	15 (25.0)	15 (50.0)	–
csDMARDs	6 (10.0)	6 (20.0)	–
tsDMARDs	3 (5.0)	3 (10.0)	–
**Vaccination**			
mRNA	58 (96.7)	30 (100.0)	28 (93.3)
mRNA + vector	2 (3.3)	0 (0.0)	2 (6.7)

### Interchangeability of capillary blood and saliva with venous blood

**Figure 3:**
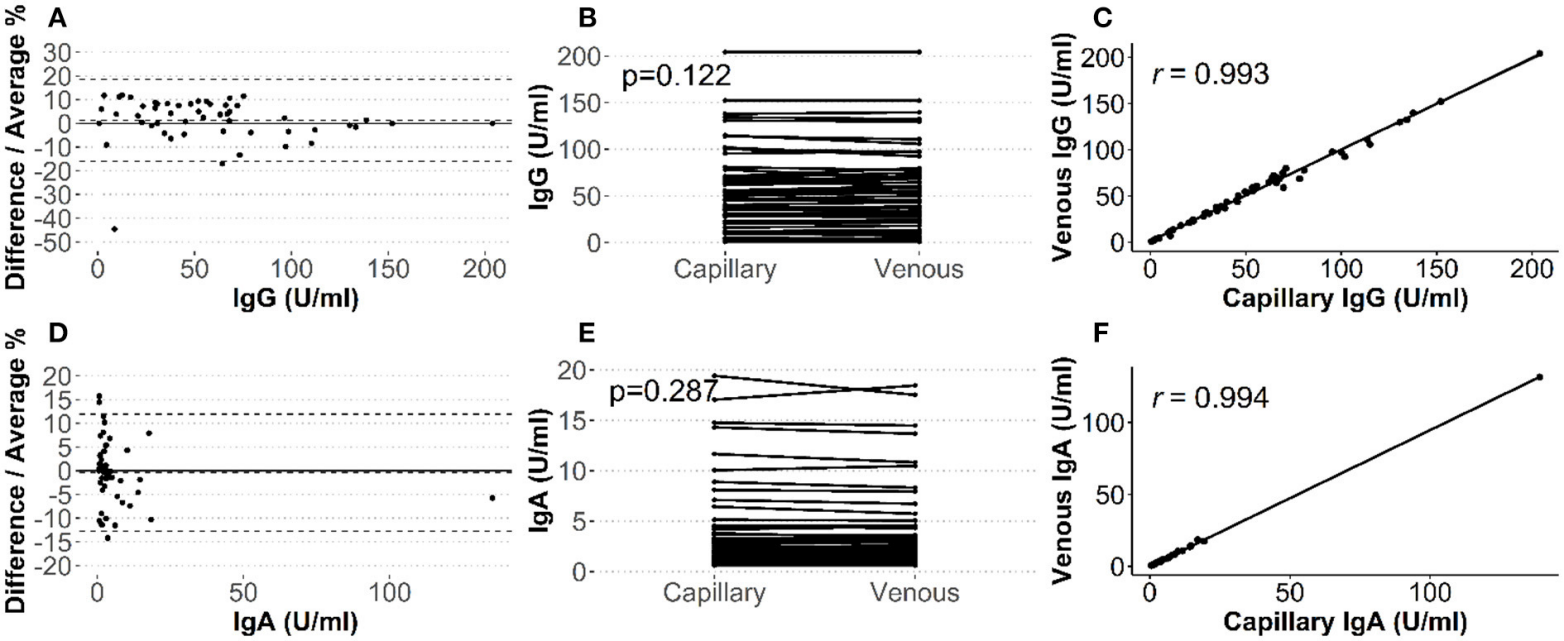
Comparison of capillary and venous antibody levels. Single dots represents individual participants. Bland-Altman plot with dashed lines representing upper and lower 95% limits of agreement **(A,D)**, paired Wilcoxon signed rank test, and Spearman's correlation analysis of measurements for IgA **(A–C)** and IgG **(D–F)**, respectively.

We observed an accuracy of 98.3% (57/58) for anti-SARS-CoV-2 IgG antibodies and 100% (58/58) accuracy for anti-SARS-CoV-2 IgA antibodies, as most of the capillary blood samples fell in the same positive and negative categories as the venous results. Only one variation was observed, where the venous serum value for anti-SARS COV-2 IgG antibodies (6.7 U/ml) was close to the equivocal range of 7 to 10 U/ml and the value measured in the capillary sample (10.5 U/ml) and was just above the cut-off of 10 U/ml. A priori criteria to demonstrate interchangeability to venous blood were also met by capillary blood-based SARS-CoV-2 IgG and IgA**. **IgG and IgA demonstrated an excellent correlation (r_s_ = 0.99), non-significant Wilcoxon signed-rank test (IgG: 0.12; IgA: 0.29), a small bias (IgG: 1.26%; IgA: −0.44%) and the majority of measurements were within a 10% difference (IgG: 86.3%; IgA: 86.3%), see Figure 3; Supplementary material 2.

The device, with which the saliva measurements were performed was not completely developed at the time of this study, and the values were not directly comparable (see Supplementary material 1).

### Usability, acceptability and pain

Usability of both self-sampling procedures was rated as excellent, with significantly higher saliva SUS total scores in both groups, resulting in total SUS scores of 95.9 ± 5.7 vs. 90.4 ± 9.7 (*p* < 0.001), see Table 2.

**Table 2:** Means and standard deviation scores for the System Usability Scale.

	**Total** **(*****n** **=*** **60)**		**Patients** **(*****n** **=*** **30)**		**Health professionals** **(*****n** **=*** **30)**	
**Questions^a^** **mean ±SD**	**Saliva**	**Tasso+**	**Saliva**	**Tasso+**	**Saliva**	**Tasso+**
1. I think I would like to use the system frequently	4.5 ± 1.1	4.4 ± 0.8	4.5 ± 1.1	4.5 ± 0.7	4.5 ± 1.1	4.3 ± 0.8
2. I found the system to be unnecessarily complex	1.1 ± 0.3	1.2 ± 0.4	1.1 ± 0.4	1.1 ± 0.3	1.0 ± 0.2	1.2 ± 0.5
3. I thought the system was easy to use	4.9 ± 0.5	4.8 ± 0.6	5.0 ± 0.2	4.9 ± 0.3	4.8 ± 0.7	4.6 ± 0.7
4. I think that I would need support of a technical person to be able to use the system	1.1 ± 0.4	1.3 ± 0.9	1.1 ± 0.5	1.3 ± 0.7	1.1 ± 0.3	1.4 ± 1.0
5. I found the various functions in the system were well integrated	4.9 ± 0.3	4.6 ± 0.7	5.0 ± 0.0	4.6 ± 0.8	4.9 ± 0.4	4.6 ± 0.6
6. I thought there was too much inconsistency in the system	1.5 ± 0.9	1.7 ± 1.0	1.3 ± 0.7	1.6 ± 1.2	1.6 ± 1.0	1.7 ± 0.9
7. I would imagine that most people would learn to use the system very quickly	5.0 ± 0.2	4.3 ± 1.0	5.0 ± 0.0	4.5 ± 0.7	4.9 ± 0.3	4.1 ± 1.1
8. I found the system very cumbersome to use	1.1 ± 0.3	1.2 ± 0.6	1.1 ± 0.3	1.1 ± 0.4	1.1 ± 0.3	1.3 ± 0.7
9. I felt very confident using the system	4.8 ± 0.7	4.6 ± 0.7	4.8 ± 0.8	4.6 ± 0.8	4.8 ± 0.6	4.6 ± 0.7
10. I needed to learn a lot of things before I could get going with the system	1.1 ± 0.2	1.2 ± 0.6	1.1 ± 0.3	1.2 ± 0.6	1.0 ± 0.2	1.2 ± 0.7
System Usability Scale total score (out of 100)	95.9 ± 5.7	90.4 ± 9.7	96.8 ± 5.0	92.1 ± 9.1	95.1 ± 6.2	88.7 ± 10.1

^*a*^Responses were scored on a five-point Likert scale: 1=strongly disagree, 5=strongly agree.

**Figure 4:**
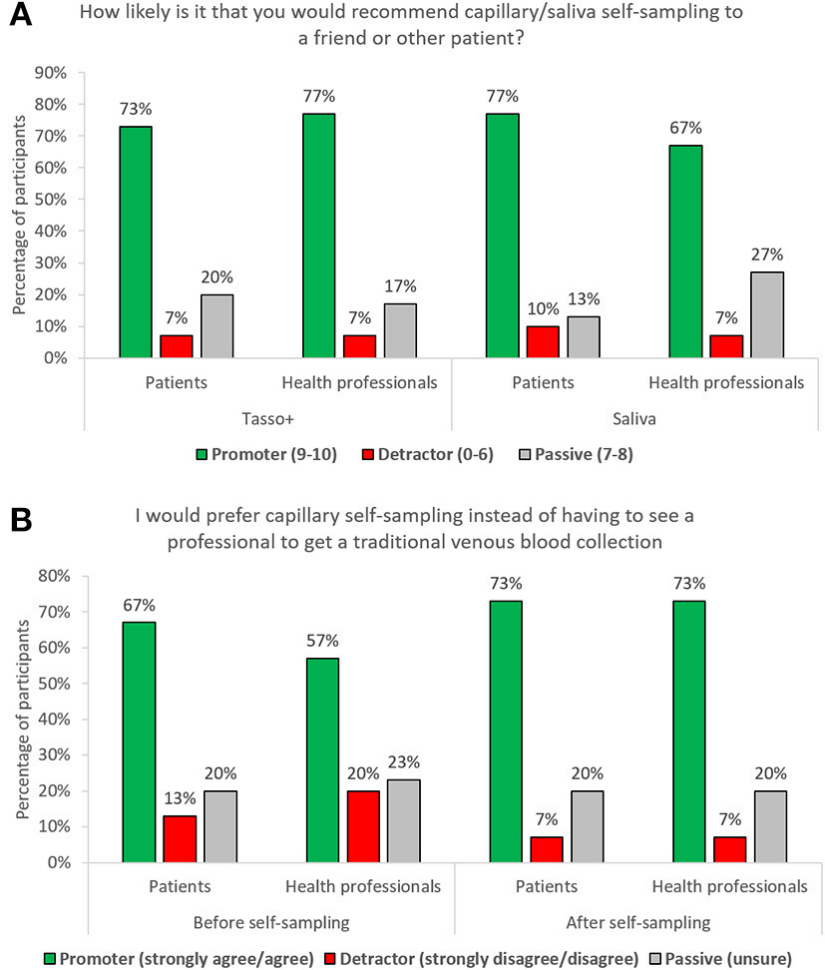
**(A)** Percentage of participants per group according to respective net promoter category and self-sampling device; **(B)** participants preference of capillary self-sampling over traditional venous blood collection.

The percentage of NPS promoters (NRS 9-10), was similar for both devices (Figure 4A), ranging between 67 and 70%, resulting in a slightly higher NPS score for capillary self-sampling: + 68 vs. + 63%. Acceptance of capillary self-sampling was generally high both in patients and HPs and even further increased after having done the procedure (Figure 4B). Furthermore, the majority in both groups preferred capillary self-sampling to professional venous blood collection (IMID: 73%; HP: 73%), see Figure 4B.

Capillary self-sampling was perceived as significantly (*p* < 0.001) less painful compared to traditional venous blood collection (IMID: 1.1 ± 0.3 vs. 2.5 ± 1.9; HP: 1.5 ± 1.2 vs. 1.9 ± 1.1). Sixty-three point three percentage and 36.7% of IMID patients perceived capillary self-sampling as less or equally painful compared to venous blood collection. In the HP group 53.3, 36.7 and 10.0% perceived capillary self-sampling as less, equally or more painful compared to venous blood collection.

### Self-sampling success rate and supervision

58/60 capillary blood samples and all 60 saliva samples were successfully collected within the first attempt.

#### Saliva self-sampling supervision

All participants except one (59/60, 98.3%) stated to have adhered to not eating or drinking 30 min prior to saliva collection. 5/60 (8.3%) participants had to be reminded to remove the cotton from the test tube and 3/60 (5.0%) needed assistance to do that. There was uncertainty among 7/60 (11.67%) participants if the small test tube could be thrown in the trash or not. 7/60 (11.67%) participants were unsure when assessing if enough saliva was collected, especially since saliva was often foamy.

#### Capillary self-sampling supervision

**Figure 5:**
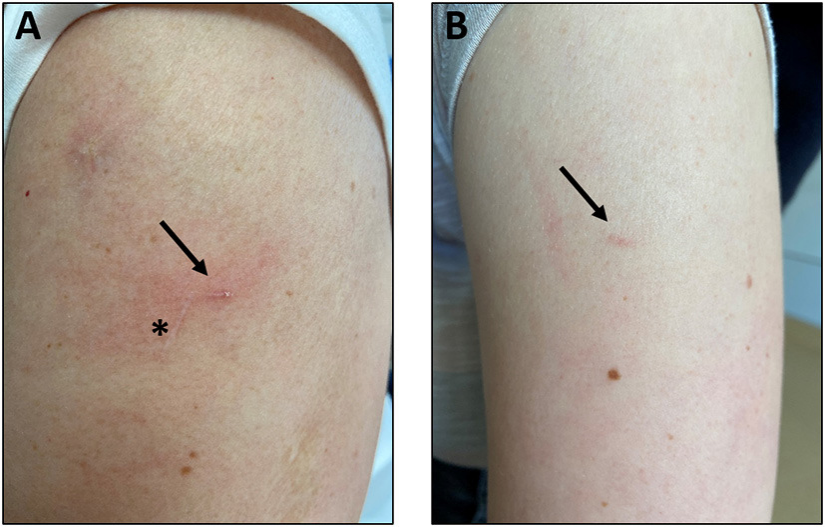
Scars (arrows) of two individual participants **(A)** 91 days and **(B)** 89 days after self-sampling. Asterisk to clarify diagonal previous unrelated scar.

One patient (1/60, 1.7%) and one HP (1/60, 1.7%) failed to collect capillary blood. Both participants stated to be in a hurry, did not pay adequate attention to the instructions and failed to adequately attach the self-sampling device. 17/60 (28.3%) participants did not follow the protocol steps (e.g. wanted to self-sample before attaching the collection tube). 11/60 (18.3%) participants had to be reminded to start the timer while applying the heat pad to the selected spot on the upper arm. Most problems occurred using the heat pad, where 2/60 (3.3%) pointed out that the heat was getting uncomfortable, and one participant stopped the application prematurely. Additionally, 4/60 (6.7%) participants did not understand how to apply the heat pad, 9/60 (15.0%) participants needed assistance with the activation of the heat pad and in 12/60 (20.0%) cases the heat pad was malfunctioning and had to be replaced. About 7/60 (11.7%) did not carry out the disinfection correctly (e.g., had to be reminded, performed too early). 5/60 (8.3%) had difficulties with removing the protective foil. Two participants accidentally teared the adhesive foil off. The device wasn't applicated properly on the selected spot on the upper arm in 4/60 (6.7%) cases. 10/60 (16.7%) participants expressed concern about the device falling off and held on to it during blood collection. After pushing the button, 7/60 (11.7%) participants would have forgotten to start the timer. Assistance for checking the filling state of the test tube was needed in 10/60 (16.7%) cases. Many participants pointed out that they would have used a mirror if they had done the self-sampling at home. The study personnel had to intervene three times when devices (still connected with collection tube) were put on a flat surface with the risk of blood spilling out. Three participants needed assistance to remove and close the test tube. One of them pointed out the lack of strength and fine motor skills in her fingers due to rheumatoid arthritis. The test tube was shaken instead of slowly turned 5/60 (8.3%) times. Three participants had to be reminded of this step. 6/60 (10.0%) participants reported problems with the healing process. Five of them developed a scar, see Figure 5. Tasso has been working on improvements to that effect.

## Discussion

In this study comparing capillary- and saliva-based self-sampling in IMID patients and HP we demonstrate that self-collection of capillary blood and saliva is feasible. Importantly, we also demonstrated that capillary blood produces interchangeable results to conventional venous blood. Participants reported high acceptance for self-sampling with a slight preference for capillary self-sampling. The majority in both groups preferred capillary self-sampling over traditional venous blood collection. Supervision of self-collection allowed the identification of pitfalls to improve the self-sampling approach.

Importantly, we were able to demonstrate the interchangeability of capillary-based anti-SARS-CoV-2 antibodies, allowing precise home-based monitoring. These results are in line with a previous study that reported high correlation despite exposing samples to extreme shipping conditions (9) using a previous upper-arm device. Brown et al. also demonstrated the feasibility of capillary self-sampling and that storage of capillary blood at room temperature for up to 7 days post sampling did not affect concordance (21). Similarly, a dried blood spot (DBS) study demonstrated accuracy using only 10 μl of blood and demonstrate the scalability of this home-based approach by conducting a population-based study with a success rate of 82% (22).

SARS-CoV-2 antibody saliva-based analysis has been validated in various populations, including children (11) and COVID-19 patients (10). Contradicting observations of agreement between saliva and serum IgG or IgA levels were reported. Isho et al. (23) described only moderate correlations while others (10, 24) observed good correlation of IgG titers against spike and nucleocapsid antigens. In this study, the values of SARS-CoV-2 spike antigen IgG and IgA antibodies in the saliva were based on a not fully developed device and showed no significant correlation with venous or capillary serum samples. While individual samples showed reasonable concordance it can be speculated that there are multiple contributors to the heterogeneity of saliva samples. Ortega et al. (25) discuss the different sources of saliva IgA (produced locally in salivary gland plasma cells) and IgG (passive diffusion from serum) as a reason for differences in the observed titers. Additionally, saliva sampling shows generally more variations compared to capillary blood because it is more dependent on instruction compliance (no eating/drinking) prior to sampling (24), varying amounts of remaining mucines and individual degrees of viscosity. Recently Campbell et al. (24) reported that salivary antibodies are stable without refrigeration or preservatives for at least 5 days and piloted a saliva collection kit that can be used *via* regular mail, yet in contrast to HIV (26), no saliva-based serology tests are currently commercially available. While many laboratory test kits for the determination of anti-SARS-CoV-2 antibodies are designed for the use with serum or plasma only, it can be speculated that assay technology specifically developed for use with saliva samples may also contribute to higher agreements in antibody titers.

Due to the greater availability of serum-based analysis devices, capillary blood will likely be easier to implement for the time being.

We observed excellent usability (SUS) of both devices and a statistically significant higher saliva SUS score. Compared to the previous RA study (8) with a mean SUS of 83.1 for the upper-arm device and 80.7 for the finger prick, we observed meaningfully higher ratings in this study for the new Tasso device 90.4 and saliva-based sampling, 95.9. Similarly we observed higher NPS ratings in this study (+68%) compared to the previous RA study (+28%). We can only speculate on the reasons for this difference. We believe that the idea of remote COVID-testing (this study) was easier to grasp as participants were already used to COVID self-sampling (antigen) compared to a more novel idea of CRP and RA-related antibody testing (RA study). We could support previous findings, that upper-arm devices are perceived as significantly less painful compared to venous blood collection (8, 27, 28). The number of patients with less pain using the capillary device compared to venous blood collection was very similar to the previous RA study (8) (63 vs. 60%). Interestingly, we were able to show that actual usage of the devices does change the level of acceptance in at least some participants. After usage the majority of participants would prefer capillary self-sampling over traditional venous collection.

58/60 (96.7%) were able to successfully collect capillary blood within first attempt. Medical education (HP) did not seem to have significant effect on success rate or correct completion of self-sampling steps. In a previous study evaluating a former version of the upper-arm device in patients with rheumatoid arthritis (RA), 16% of the patients needed a second attempt and 4% of patients failed to carry out the procedure (8). In a similar study investigating participants with a prior SARS-CoV-2 infection 7% needed a second attempt and no patients failed to perform self-sampling (9). In the same study 32% requested help. Interestingly, in the previous study the most frequent reason for assistance with the device was help to activate it by pressing the button. In contrast to the previous study we tried to standardize the procedure to increase local blood flow and chose heat-pads instead of skin rubbing. The chosen heat-pads devices failed to work multiple times and as we only gave oral instructions to participants, using the heat-pad was the greatest challenge. Additionally, participants needed help to remove the protective film from the self-adhesive patch and accidentally removed the patch itself.

This study has several limitaitons, including the small sample size. A main limitation is that we did not explore the ultimate goal of a home-based remote study. This risk-adverse study setting was chosen, so that correct usage could closely be monitored and study personnel could physically intervene in case of danger. In a next study we want to explore the at-home scenario and provide on-demand help with videoconsultations, as we did not see any major dangers in this study. A home-based study could also involve caring personnel, in case patients cannot use the devices alone. We could gain valuable user feedback regarding usability and acceptance of capillary and saliva sampling. The matched cohorts, including different age groups and diseases are a strength of this study allowing to assess the benefit of having medical training (HP). Usage of a validated composite approach (7) to investigate interchangeability and detailed observation of correct self-sampling execution represent strengths of this study.

## Conclusion

Self-collection of capillary blood and saliva is feasible and safe and could facilitate access to antibody testing of the general public. The interchangeability and high acceptance of capillary blood self-sampling enable flexible and convenient vaccine immunogenicity monitoring.

## Data availability statement

The raw data supporting the conclusions of this article will be made available by the authors, without undue reservation.

## Ethics statement

The studies involving human participants were reviewed and approved by the Ethics Committee of the Medical Faculty of the University of Erlangen-Nürnberg, Germany. The patients/participants provided their written informed consent to participate in this study.

## Author contributions

CS and JK wrote the draft manuscript. CS, JK, and E-TG performed the statistical analysis. All authors contributed to the article and approved the submitted version.

## Funding

The study was supported by the Deutsche Forschungsgemeinschaft (DFG–FOR 2886 PANDORA - Z/B01/A03/Z/C1 to JK, GK, GS, AK, and DS) and Thermo Fisher Scientific (Freiburg, Germany). This project has received funding from the Innovative Medicines Initiative 2 Joint Undertaking (grant agreement No. 101007757, HIPPOCRATES). This study received funding from Thermo Fisher Scientific. The funder had the following involvement with the study: Provision of study materials, sample measurements and revision of the manuscript. The funder was not involved in the decision to submit the study for publication.

## Conflict of interest

Authors EV and LS were employed by Thermo Fisher Scientific Inc. The remaining authors declare that the research was conducted in the absence of any commercial or financial relationships that could be construed as a potential conflict of interest. The authors declare that this study received funding from Thermo Fisher Scientific. The funder had the following involvement with the study: Provision of study materials, sample measurements and revision of the manuscript. The funder was not involved in the decision to submit the study for publication.

## Publisher's note

All claims expressed in this article are solely those of the authors and do not necessarily represent those of their affiliated organizations, or those of the publisher, the editors and the reviewers. Any product that may be evaluated in this article, or claim that may be made by its manufacturer, is not guaranteed or endorsed by the publisher.
