# Supplementary material for: Self-collection of capillary blood and saliva to determine COVID-19 vaccine immunogenicity in patients with immune-mediated inflammatory diseases and health professionals
Source: Front Public Health. 2022 Oct 14;10:994770. doi: 10.3389/fpubh.2022.994770 (PMC9616117; doi:10.3389/fpubh.2022.994770)
Supplement: Supplementary file 1 [file Data_Sheet_1.PDF]

Table S1. Raw data for venous and saliva IgG and IgA values.

Patient	Saliva EliA SARS-CoV-2-Sp1 IgG RU (response units)	Saliva EliA SARS-CoV-2-Sp1 IgA RU (response units)	Venous EliA SARS-CoV-2-Sp1 IgG (U/ml)	Venous EliA SARS-CoV-2-Sp1 IgA (U/ml)
1	197.25	422.29	69.79	2.93
2	180.43	413.15	97.61	1.81
3	300.97	310.37	68.31	0.66
4	95.26	589.50	54.40	<0.6
5	292.05	749.64	58.84	17.51
6	1371.21	653.88	43.54	3.31
7	24.35	502.18	9.84	<0.60
8	14.86	520.35	30.67	3.60
9	77.42	310.19	77.57	6.73
10	289.22	1369.11	22.85	1.40
11	83.15	651.96	55.26	2.84
12	223.02	1357.20	96.77	3.31
13	59.59	582.52	24.09	1.41
14	523.38	213.72	129.72	<0.60
15	78.52	515.76	>204.00	8.32
16	738.62	4699.96	74.80	5.74
17	12277.76	7849.10	>204.00	131.35
18	256.51	182.43	92.34	1.02
19	311.63	2336.87	139.64	2.93
20	11.32	349.49	<0.70	3.02
21	632.91	565.29	>204.00	3.17
22	1053.79	735.86	>204.00	18.45
23	111.61	297.54	105.71	0.71
24	56.49	98.56	68.44	1.60
25	5508.24	1080.16	>204.00	1.61
26	50.18	194.73	50.05	14.48
27	39.52	222.29	38.59	0.60
28	37.37	441.13	53.38	4.33
29	102.75	292.30	33.50	2.37
30	249.17	4239.36	3.44	2.60

31	607.50	439.73	>204.00	13.66
32	657.98	4363.99	132.25	0.75
33	127.01	626.62	27.63	1.16
34	55.96	238.94	33.66	2.33
35	52.92	444.63	30.80	0.96
36	51.92	957.31	21.07	4.47
37	283.31	166.15	110.67	4.52
38	267.02	440.61	64.35	1.16
39	366.76	2004.70	63.84	10.83
40	31.84	227.47	4.26	<0.60
41	313.71	642.09	43.34	2.47
42	607.69	820.55	68.27	3.02
43	206.82	542.18	>204.00	7.93
44	124.67	2186.72	9.76	2.41
45	91.79	566.37	6.67	1.18
46	317.67	1616.13	71.89	3.20
47	184.75	250.28	79.74	2.32
48	51.24	974.70	31.00	5.06
49	198.99	333.53	60.43	<0.60
50	38.10	803.29	11.68	1.96
51	191.31	305.66	152.07	1.36
52	20.23	69.35	13.40	<0.60
53	76.56	779.78	36.57	1.26
54	28.95	2784.91	1.97	<0.60
55	43.55	509.96	58.74	2.92
56	120.71	616.09	32.06	1.30
57	312.68	275.95	68.66	2.16
58	54.59	302.20	45.48	4.41
59	262.56	414.24	37.46	0.70
60	30.71	277.49	17.93	10.48

Table S2. Interchangeability analysis of self-sampled capillary blood following the methodology of Nwankwo et al. (7)

Test	N° of paired samples	Mean of difference	SEM	95% CI	P-Value (Wilcoxon Signed-Rank Test)	Correlation		Bland-Altman				Equivalence Range	Interchangeably in clinical setting based on Wilcoxon Signed-Rank Test p>0.05	Interchangeably in clinical setting based on rho>0.8	Interchangeably in clinical setting based on small Bias OR max 10% difference between tests	Point scoring for clinical acceptability
						r_s[1]	95% CI	Bias (%)	SD of bias (%)	95% limits of agreement (%)						
										From	To					
**IgG**	58	0.35	0.50	-0.65 to 1.34	0.12	0.99***	0.97 to 1.0	1.26	8.87	-16.12	18.63	7-10 U/ml	Yes	Yes	Yes	3
**IgA**	58	-0.20	0.14	-0.48 to 0.08	0.29	0.99***	0.98 – 1.0	-0.44	6.31	-12.79	11.92	7-10 U/ml	Yes	Yes	Yes	3

Statistical significance: * < 0.05, ** < 0.01, *** <0.001. [1]Spearman Correlation
